# Genomic prediction of genetic merit using LD-based haplotypes in the Nordic Holstein population

**DOI:** 10.1186/1471-2164-15-1171

**Published:** 2014-12-23

**Authors:** Beatriz CD Cuyabano, Guosheng Su, Mogens S Lund

**Affiliations:** Center for Quantitative Genetics and Genomics, Department of Molecular Biology and Genetics, Aarhus University, Kragujevac, Denmark

**Keywords:** Genomic prediction, High-density data, Haplotypes, Linkage disequilibrium

## Abstract

**Background:**

A haplotype approach to genomic prediction using high density data in dairy cattle as an alternative to single-marker methods is presented. With the assumption that haplotypes are in stronger linkage disequilibrium (LD) with quantitative trait loci (QTL) than single markers, this study focuses on the use of haplotype blocks (haploblocks) as explanatory variables for genomic prediction. Haploblocks were built based on the LD between markers, which allowed variable reduction. The haploblocks were then used to predict three economically important traits (milk protein, fertility and mastitis) in the Nordic Holstein population.

**Results:**

The haploblock approach improved prediction accuracy compared with the commonly used individual single nucleotide polymorphism (SNP) approach. Furthermore, using an average LD threshold to define the haploblocks (*LD*≥0.45 between any two markers) increased the prediction accuracies for all three traits, although the improvement was most significant for milk protein (up to 3.1 *%* improvement in prediction accuracy, compared with the individual SNP approach). Hotelling’s t-tests were performed, confirming the improvement in prediction accuracy for milk protein. Because the phenotypic values were in the form of de-regressed proofs, the improved accuracy for milk protein may be due to higher reliability of the data for this trait compared with the reliability of the mastitis and fertility data. Comparisons between best linear unbiased prediction (BLUP) and Bayesian mixture models also indicated that the Bayesian model produced the most accurate predictions in every scenario for the milk protein trait, and in some scenarios for fertility.

**Conclusions:**

The haploblock approach to genomic prediction is a promising method for genomic selection in animal breeding. Building haploblocks based on LD reduced the number of variables without the loss of information. This method may play an important role in the future genomic prediction involving while genome sequences.

## Background

Genomic prediction for important dairy traits such as production, fertility and health traits using single nucleotide polymorphisms (SNPs), has been widely explored and applied in animal breeding. After genomic prediction methods using moderate marker data (≈50 *k*) were introduced [1], they have become the topic of interest for several studies in animal breeding. When high density (HD) marker data (770 *k*) became available, the accuracy of genomic prediction was expected to improve [2] as a result of an increased degree of linkage disequilibrium (LD) between the SNPs and the underlying quantitative trait loci (QTL) associated with the genetic variation in the traits of interest.

So far this expectation has not been realized, because predictions using HD data have not shown very significant improvements [3–5] over similar predictions based on moderate density data. Currently, genotypic data is available for hundreds of Nordic Holstein bulls that were genotyped with 770 *k* SNP chips which raised the question of how this data can be used to improve the accuracy of genomic predictions. A further challenge is to process the large number of variables so that genomic predictions can be performed as efficiently as possible.

It has been reported that HD genotypic data for individual animals genotyped with the current Illumina 54 *k* bovine chip can be imputed accurately to 770 *k* using data from a group of representative animals that were genotyped with a HD marker chip with appropriate methods [6, 7]. In dairy cattle, an imputation method was used to generate a larger data set with more animals for genomic prediction of genetic merit for young candidates bulls, which greatly improved the accuracy of genomic prediction compared with the accuracy based on the conventional pedigree index [8, 9].

Haplotypes have been used extensively in human genetics research [10–14] and, in animal breeding studies, haplotypes have been used for the genomic prediction of breeding values [15–20]. However, because the haplotypes used in previous studies were not based on HD data, there was no need to reduce the number of predictor variables.

An important advantage of haplotypes over single SNP markers is their higher ability to identify mutations. In animal breeding studies, SNPs are commonly bi-allelic and even when mutations have occured it is possible that the allele frequencies remain (almost) unaltered. However, when haplotypes were analysed, mutations in different loci tended to cause major changes in the haplotype frequencies [11]. Thus, a QTL that is not in complete LD with any individual bi-allelic SNP marker may be in complete LD with a multi-marker haplotype.

When building haplotype blocks (haploblocks) various questions needed to be addressed including in which genomic regions the haploblocks should be defined and how many SNP markers should one haploblock contain. There was also the concern that building haploblocks would increase the number of explanatory variables because, by randomly grouping SNPs, the maximum number of variants would increase drastically. An efficient method that has been used to build haploblocks in a way that can reduce the number of explanatory variables without losing the information provided by the HD marker map, is to use LD to define where haploblocks start and end in the genome [13]. Some authors have defined haploblocks for genomic predictions by setting windows with a fixed number of SNPs to be placed together as a haploblock [17, 19], or by considering the first locus only, out of ten consecutive loci in genomic evaluation [20]. By setting a minimum amount of LD between SNP markers they can be grouped into haploblocks that do not have a fixed length (number of SNPs) and because of relatively strong LD, the number of variants per haploblock is reduced considerably, compared with when haploblocks are defined by a fixed number of physically close SNPs.

Two hypothesis are tested in this study. One hypothesis is that LD-based haploblocks can achieve a higher genomic prediction accuracy than the widely used individual SNP approach. The other hypothesis is that LD-based haploblocks, which allows a non-random grouping of SNPs, reduce the number of explanatory variables required for the predictions.

## Methods

### High-density and phenotypic data

The complete data set that was used in this study for the genomic predictions consisted of 5,214 bulls born between 1974−2008 from the Nordic Holstein population. The marker data that was obtained using the Illumina 54 *k* bovine SNP chip was imputed to HD genotypes using the Beagle package [21] and the 557 HD genotyped reference bulls in the EuroGenomics project [22]. After the imputation, the HD data was edited to remove SNPs with a minor allele frequency (MAF) lower than 0.01; markers in complete LD with adjacent markers were also removed [6]. After editing, a total of 492,057 SNPs remained in the imputed HD data. The whole data set was divided into training and test subsets by a cut-off birth date for the bulls, of 1 of October, 2001. The sizes of the training and test data sets are presented in Table 1.

**Table 1:** **Size of training and test data sets used in the genomic prediction models**

	Protein	Fertility	Mastitis
Train	3,003	3,037	3,006
Test	1,395	1,378	1,491
Total	4,398	4,415	4,497

Three economically important index traits (milk protein, fertility and mastitis) were tested in this study. The phenotypic values used for the genomic predictions were de-regressed proofs (DRP) that were derived from the estimated breeding values (EBV) and from the effective daughter contributions [23, 24].

### Animal ethics

The phenotypic data was collected from routine records of dairy cattle farms. Genotyped animals used in this work were the progeny-tested bulls, and the semen samples for genotyping were obtained from routine bull semen collection. Therefore, no ethical approval was necessary.

### Haplotyping method

The LD-based haploblocks were built separately for each chromosome. A group of SNPs was defined as a haploblock if the LD between **every two** SNPs in the group was greater than or equal to a certain threshold *d*. This method ensured that the markers that were physically close and presented a minimum defined amount of LD were placed in the same haploblock. This LD structure allowed non-randomly associated SNPs to be grouped together in one haploblock, which reduced the number of variants in each haploblock and limited the number of explanatory variables required for the genomic predictions.

Three common pairwise LD measures have been used, *D*, *r*^2^ and *D*^′^ [25, 26]. The *r*^2^ and *D*^′^ measures are both standardized to be between zero and 1, and are less dependent on the frequencies of individual alleles than *D*. The closer *r*^2^ or *D*^′^ are to zero, the less the LD between the two SNP loci. In the present study, the *D*^′^ measure was used to build the haploblocks.

Because *D*^′^ is computed by dividing the minimum allele frequency for a pair of markers, it generates higher LD measures at loci with low allele frequencies than *r*^2^. In other words, at loci with low allele frequencies, *r*^2^ is more sensitive to LD than *D*^′^ [27]. A preliminary study was made for predictions using haploblocks built with both *D*^′^ and *r*^2^, and no significant difference on prediction reliabilities was observed. Because one of the aims of the present study was to use haploblocks also to contribute to variable reduction, *D*^′^ was chosen so that a lower rate of non-blocked SNPs was obtained and haploblocks that were too short (*e.g.* with only two or three SNPs) were avoided.

Different LD thresholds *d* were considered, from more relaxed to very strict as 0.25, 0.35, 0.45, 0.55, 0.65 and 0.75. For two bi-allelic loci with alleles *A*_1_/*A*_2_ and *B*_1_/*B*_2_, *D*^′^ is calculated by:
1

where .

As mentioned previously, this measure is standardized between zero and 1, where zero indicates no LD and 1 indicates complete LD between loci.

**Figure 1:**
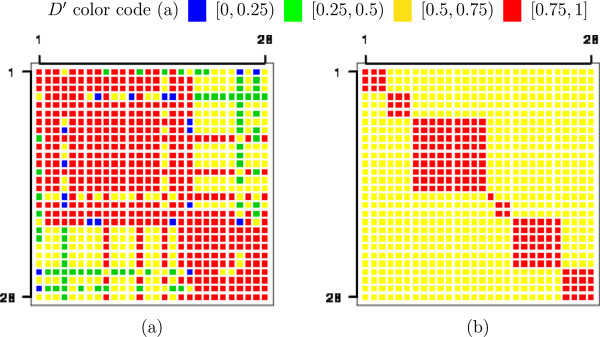
**Toy example of haploblocks built when then LD threshold was** ***D*** ^***′***^ ***≥0.75*** **.** **(a)** Pairwise LD heat map. The color code indicates the amount of LD between two SNPs. **(b)** Outlined haploblocks based on the LD heat map. The LD between every two SNPs agrees with the threshold *D* ^′^≥0.75.

A toy example that illustrates how the haploblocks were built when *D*^′^≥0.75 was set as the treshold is presented in Figure 1. First the pairwise LD between every two SNPs from the genome map was calculated, then the haploblocks were defined using the criterion previously described.

### Genomic prediction models

Genomic predictions were performed for milk protein, fertility and mastitis, because they represent the most important trait groups defined in breeding goals. Two models, a best linear unbiased prediction (BLUP) model and a Bayesian mixture model, that included the haplotypes/SNP effect and the remaining polygenic effect were used for the predictions.

These models were used for predictions with a) all the SNPs, b) the haploblocks and the non-blocked SNPs (*i.e.* single SNPs that were not placed in any block because they displayed very little LD with other SNPs), and c) the haploblocks only; the last two (b and c) with the six different *D*^′^ thresholds. This adds up to a total of 13 scenarios per trait. The genomic predictions for each of these scenarios were analyzed and compared.

The BLUP and Bayesian mixture models were executed using a Bayesian approach implemented in the BayZ package [28], running a single chain with 50,000 cycles where the first 20,000 cycles were taken as the burn-in of the chain.

#### BLUP model

The BLUP model was described as:
2

where ***y*** is the vector containing the DRP of the reference bulls, *μ* is a general mean, ***M*** is the SNP/haploblock matrix, ***g*** are the additive SNP/haploblock effects, ***Z*** is the incidence matrix linking ***a*** to ***y***, ***a*** are the residual polygenic additive genetic effects, and ***ε*** are the model errors. All the parameters in the model were assumed to have the following prior distributions:
3

where ***A*** is a genetic relationship matrix constructed according to the pedigree. ***D*** is a diagonal matrix with *d*_*ii*_=1/*w*_*i*_ and  [29, 30], *w*_*i*_ is a weighting factor that accounts for heterogeneous residual variances caused by differences in , the reliability of the *i*−*th* DRP [3].

The SNP/haploblock variables can have values equal to 0,1 or 2. For the individual SNP approach, ***M*** is *n*×*p* (*n*= number of animals, *p*= number of marker loci), in which *m*_*ij*_=0 means that for the *i*−*th* individual, neither of the two allele copies (paternal and maternal) in the *j*−*th* SNP is the allele with minor frequency, *m*_*ij*_=1 means that one of the copies is of the allele with minor frequency and *m*_*ij*_=2 means that both copies are of the allele with minor frequency. For the haploblock approach there may be more than one variable for each haploblock, because each haploblock may have more than two variants. In this case, ***M*** is *n*×*q* (*n*= number of animals, *q*= total number of haploblock variables), in which *m*_*ij*_∈{0,1,2} means how many copies of the haploblock variant represented by the *j*−*th* column, are present in the *i*−*th* animal.

#### Bayesian mixture model

The mixture model was described by the same equation as the BLUP model,
4

However, there is a difference here in the distribution of ***g***, the additive SNP/haploblock effects. The mixture model [31], was used to facilitate the mixing of the Markov Chain Monte Carlo (MCMC) on the HD marker data, and is an extended version of previously proposed methods [32, 33]. All parameters in the model were assumed to have the following prior distributions:
5

The variances and the effects were estimated simultaneously. The mixing proportions *π*_*k*_ were fixed as *π*_1_=0.889, *π*_2_=0.1, *π*_3_=0.01 and *π*_4_=0.001, and their uniformly distributed variances were constrained as: . Because the highest proportion of the effects shows the smallest variance, the normal distribution that weights this proportion has the highest probability of being close to zero.

### Analysis and comparison of genomic predictions

The genomic estimated breeding values (GEBV) obtained from the prediction models were calculated as,
6

and the performances of the two models with all the marker data sets for each trait were compared using the prediction reliability . The bias of prediction was assessed using a regression coefficient *b* of DRP on the GEBV [3].

The  coefficient was calculated as the squared correlation between DRP and GEBV corrected for the reliability of average DRP,  [30],
7

To check whether there was indeed significant difference between the SNP approach (taken as the reference) and the haploblock approach, the prediction reliabilities were compared using Hotelling’s test [34]. It should be noted that a  comparison is equivalent to a *Cor*(*DRP*,*GEBV*[model1])=*Cor*(*DRP*,*GEBV*[m o d el2]) comparison. For *ρ*_*GEBV*,*i*_=*Cor*(*DRP*,*GEBV*[modeli]) and *ρ*_*ij*_=*Cor*(*GEBV*[modeli],*GEBV*[modelj]) the statistic used to test whether *H*_0_:*ρ*_*GEBV*,*i*_=*ρ*_*GEBV*,*j*_ or *H*_0_:*ρ*_*GEBV*,*i*_≥*ρ*_*GEBV*,*j*_ was true, was defined as follows,
8

where *r* is the observed correlation, *n* the number of observations and *D* is the determinant of the correlation matrix between *DRP* and *GEBV* for models *i* and *j*. If *P*(*T*≥*t*)≤*α*, then the hypothesis (*H*_0_) is rejected. Hence, correlations were considered to be statistically different with a significance level *α*.

## Results

The total number of haploblocks and the related variables for each *D*^′^ threshold, obtained from the HD marker data with 492,057 SNPs are presented in Table 2. The number of haploblock variables did not increase drastically when the *D*^′^ threshold was made more strict, and the number of variables increased at a slower rate than the number of haploblocks that were built. These findings indicate that the use of haploblocks with HD data can reduce the number of explanatory variables in the two models by up to 30*%* (*D*^′^≥0.25).

**Table 2:** **Total number of haploblocks, related variables, and non-blocked SNPs from HD data with 492,057 SNPs**

***D*** ^***′***^	Haploblocks	Variables	Non-blocked SNPs
0.25	55,513	338,460	3,513
0.35	62,309	346,938	5,399
0.45	68,318	353,221	7,744
0.55	73,928	358,461	10,280
0.65	79,154	362,455	13,207
0.75	84,634	366,167	16,812

**Figure 2:**
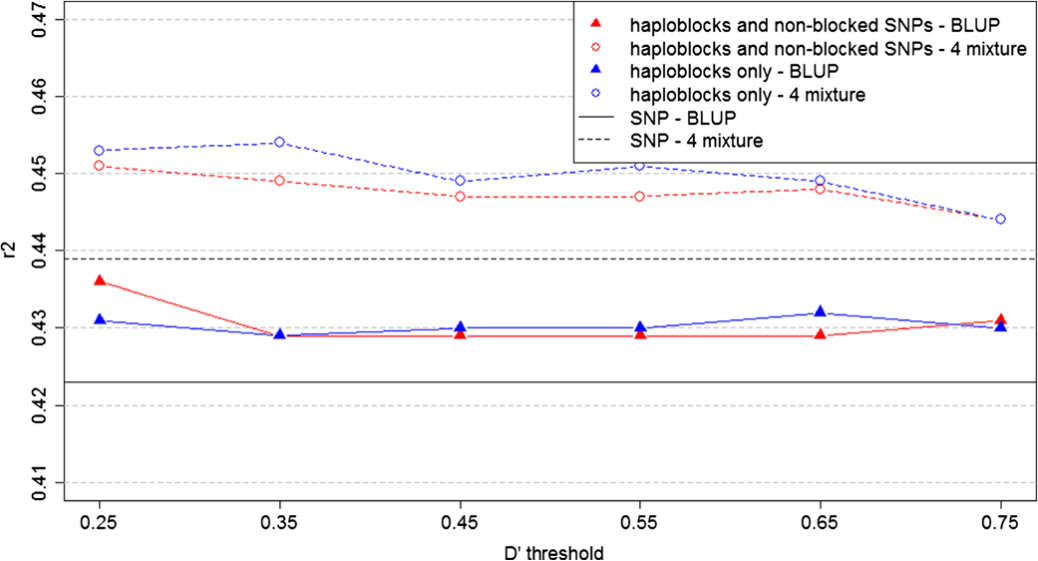
**Prediction reliabilities**  **for milk protein for BLUP and Bayesian mixture models with different** ***D*** ^***′***^ **thresholds.** This figure presents the prediction reliabilities of the models performed for milk protein. Black lines, individual SNP approach; red lines, haploblock approach with non-blocked SNPs; blue lines, haploblock approach without non-blocked SNPs. Continuous lines indicate the BLUP models; dashed lines indicate the mixture models.

**Figure 3:**
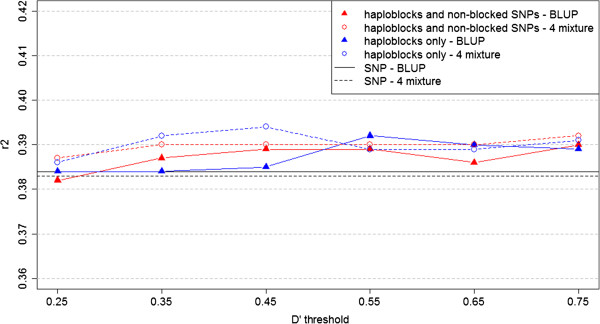
**Prediction reliabilities**  **for fertility for BLUP and Bayesian mixture models with different** ***D*** ^***′***^ **thresholds.** This figure presents the prediction reliabilities of the models performed for fertility. Black lines, individual SNP approach; red lines, haploblock approach with non-blocked SNPs; blue lines, haploblock approach without non-blocked SNPs. Continuous lines indicate the BLUP models; dashed lines indicate the mixture models.

**Figure 4:**
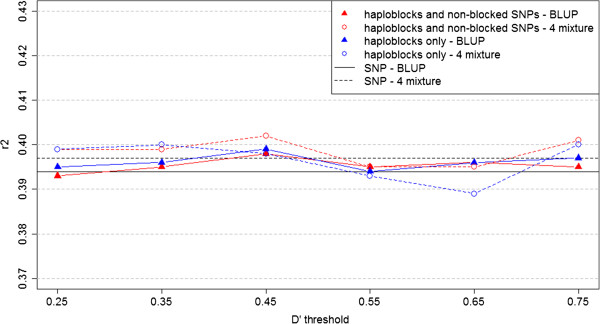
**Prediction reliabilities**  **for mastitis for BLUP and Bayesian mixture models with different** ***D*** ^***′***^ **thresholds.** This figure presents the prediction reliabilities of the models performed for mastitis. Black lines, individual SNP approach; red lines, haploblock approach with non-blocked SNPs; blue lines, haploblock approach without non-blocked SNPs. Continuous lines indicate the BLUP models; dashed lines indicate the mixture models.

The prediction reliabilities  for the three traits of interest were compared for both the BLUP and mixture models, using the HD marker data for both the individual SNPs and haploblock approaches and comparing the different *D*^′^ thresholds (Figures 2, 3 and 4). The two models seemed to produce superior prediction reliabilities for the milk protein and fertility traits using the haploblock approach.

To test if the observed differences were statistically significant and to verify if genomic prediction was improved using haploblocks, the reliabilities of the genomic predictions obtained using the two models were compared using Hotelling’s test (8). The p-values for each these comparisons are displayed in Tables 3, 4 and 5.

**Table 3:** **P-values of the Hotelling’s t-test comparing the prediction reliabilities**  **obtained with the BLUP and Bayesian mixture models**

						Haploblocks and						
		Individual SNPs				non-blocked SNPs				Haploblocks only		
***D*** ^***′***^		Prot.	Fert.	Mast.		Prot.	Fert.	Mast.		Prot.	Fert.	Mast.
—		0.034	0.624	0.254		—	—	—		—	—	—
0.25		—	—	—		0.007	0.008	0.111		0.000	0.234	0.224
0.35		—	—	—		0.000	0.108	0.279		0.000	0.001	0.236
0.45		—	—	—		0.001	0.408	0.277		0.000	0.000	0.542
0.55		—	—	—		0.000	0.239	0.414		0.000	0.900	0.532
0.65		—	—	—		0.000	0.030	0.571		0.001	0.689	0.892
0.75		—	—	—		0.008	0.284	0.208		0.011	0.172	0.201

**Table 4:** **P-values of the Hotelling’s t-test comparing the prediction reliabilities**  **obtained with the individual SNPs and Haploblocks approaches using BLUP model**

		Haploblocks and						
		non-blocked SNPs				Haploblocks only		
***D*** ^***′***^		Prot.	Fert.	Mast.		Prot.	Fert.	Mast.
0.25		0.004	0.701	0.609		0.036	0.475	0.317
0.35		0.139	0.249	0.316		0.183	0.459	0.246
0.45		0.124	0.094	0.082		0.070	0.390	0.087
0.55		0.139	0.104	0.391		0.101	0.017	0.423
0.65		0.177	0.264	0.237		0.020	0.050	0.218
0.75		0.041	0.040	0.328		0.063	0.075	0.175

**Table 5:** **P-values of the Hotelling’s t-test comparing the prediction reliabilities**  **obtained with the individual SNPs and Haploblocks approaches using Bayesian mixture model**

		Haploblocks and						
		non-blocked SNPs				Haploblocks only		
***D*** ^***′***^		Prot.	Fert.	Mast.		Prot.	Fert.	Mast.
0.25		0.002	0.193	0.292		0.000	0.279	0.270
0.35		0.006	0.076	0.355		0.000	0.028	0.236
0.45		0.010	0.054	0.144		0.003	0.007	0.402
0.55		0.008	0.045	0.585		0.000	0.082	0.711
0.65		0.004	0.081	0.652		0.001	0.086	0.961
0.75		0.052	0.009	0.236		0.048	0.022	0.163

*H*_0_ was rejected (reliabilities were taken to be different) if the p−value≤0.05 in the Hotelling’s test. If p−value∈(0.05,0.15), the indication that the models may have different reliabilities were strong, however the information in the data sets was not sufficient enough to confirm this assumption.

In general, the mixture model produced better predictions than the BLUP model, and this was very clear in the predictions for milk protein. For predicting fertility with the individual SNP approach, there were no major differences between the prediction reliabilities obtained from the two prediction models. However, for the predictions for fertility with the haploblock approach, the mixture model performed better than the BLUP model, when the haploblocks were built considering a low *D*^′^ threshold. A similar result was observed for the predictions for mastitis using the haploblock approach, and the Bayesian mixture model also performed better than the BLUP model when predicting mastitis with the individual SNP approach.

In general, the haploblock approach was better than the individual SNPs approach for predicting milk protein; however, the haploblock approach was better only for some specific *D*^′^ thresholds, and most visible for the Bayesian mixture model, for predicting the other two traits.

While the improvement in prediction reliability for the milk protein and fertility traits was statistically significant using haploblocks rather than individual SNPs, for mastitis the improved prediction reliability was observed only when *D*^′^≥0.45.

The regression coefficients *b* of DRP on GEBV for the BLUP and Bayesian mixture models are presented in Tables 6 and 7, respectively. These results suggest that prediction bias is similar in the two models because the regression coefficients are similar.

**Table 6:** **Regression coefficient** ***b*** **of DRP on GEBV for BLUP models**

						Haploblocks and						
		Individual SNPs				non-blocked SNPs				Haploblocks only		
***D*** ^***′***^		Prot.	Fert.	Mast.		Prot.	Fert.	Mast.		Prot.	Fert.	Mast.
—		0.878	1.059	1.069		—	—	—		—	—	—
0.25		—	—	—		0.834	1.067	1.047		0.889	1.070	1.049
0.35		—	—	—		0.884	1.043	1.049		0.882	1.083	1.047
0.45		—	—	—		0.880	1.057	1.054		0.891	1.054	1.051
0.55		—	—	—		0.895	1.058	1.048		0.890	1.073	1.064
0.65		—	—	—		0.884	1.061	1.060		0.881	1.095	1.052
0.75		—	—	—		0.883	1.028	1.063		0.881	1.054	1.071

**Table 7:** **Regression coefficient** ***b*** **of DRP on GEBV for mixture models**

						Haploblocks and						
		Individual SNPs				non-blocked SNPs				Haploblocks only		
***D*** ^***′***^		Prot.	Fert.	Mast.		Prot.	Fert.	Mast.		Prot.	Fert.	Mast.
—		0.878	1.048	1.026		—	—	—		—	—	—
0.25		—	—	—		0.864	1.050	1.025		0.868	1.050	1.017
0.35		—	—	—		0.884	1.040	1.022		0.867	1.053	1.026
0.45		—	—	—		0.873	1.057	1.021		0.864	1.049	1.021
0.55		—	—	—		0.858	1.048	1.002		0.876	1.073	0.981
0.65		—	—	—		0.874	1.042	1.000		0.869	1.061	1.003
0.75		—	—	—		0.866	1.062	1.040		0.866	1.061	1.024

The percentage of the top 10 selected elite bulls that is equivalent when using either an individual SNP or a haploblock approach is presented in Table 8 for BLUP and in Table 9 for the Bayesian mixture model. Using the BLUP model for protein, the overlap between the top 10 bulls selected using the individual SNP approach and the haploblock approach with different D0 thresholds was 74.3−78.6*%*. When the Bayesian mixture model was used, this proportion was 65.7−72.9*%*. When selecting the bulls for fertility the proportion was 38.6−46.4*%* using the BLUP model and 37.9−50.7*%* when using the Bayesian mixture model. This shows that using haploblocks approach instead of individual SNPs, both increase the reliability of genomic predictions in these two traits and result in a considerable difference in the elite bulls selected. The proportions of equivalent top 10 selected elite bulls observed in mastitis were 77.1−82.1*%* when using BLUP model and 88.6−96.4*%* when using the Bayesian mixture model. This indicated that there was not so clear differences in the selected bulls when using the haploblock approach instead of individual SNPs. Furthermore, pairwise comparisons of the bulls selected by the haploblock approaches for the different *D*^′^ thresholds showed that, for protein and fertility, at least 85*%* of the top 10 elite bulls are always the same for this approach, regardless of the *D*^′^ threshold. For mastitis this proportion was of 95.7*%*. This indicated that the *D*^′^ threshold had a minor influence on the top ranking of animals by the genomic prediction models.

**Table 8:** **Proportion (%) of top 10 elite bulls that are selected by both approaches for BLUP models**

		Haploblocks and						
		non-blocked SNPs				Haploblocks only		
***D*** ^***′***^		Prot.	Fert.	Mast.		Prot.	Fert.	Mast.
0.25		74.3	38.6	77.1		75.0	40.0	80.7
0.35		76.4	42.1	77.9		77.1	45.7	82.1
0.45		75.7	44.3	80.7		75.0	45.0	79.3
0.55		75.0	42.9	80.7		76.4	45.7	79.3
0.65		78.6	43.6	79.3		77.1	42.9	77.9
0.75		75.0	46.4	81.4		75.7	52.9	78.9

**Table 9:** **Proportion (%) of top 10 elite bulls that are selected by both approaches for mixture models**

		Haploblocks and						
		non-blocked SNPs				Haploblocks only		
***D*** ^***′***^		Prot.	Fert.	Mast.		Prot.	Fert.	Mast.
0.25		70.7	38.6	92.1		70.0	43.6	88.6
0.35		72.9	37.9	90.0		69.3	50.7	90.7
0.45		70.0	50.0	92.1		69.3	41.4	90.0
0.55		70.0	45.0	93.6		71.4	43.6	88.6
0.65		65.7	44.3	91.4		71.4	47.9	92.9
0.75		71.4	42.9	95.0		67.1	45.7	96.4

## Discussion

Comparisons of the predictions reliabilities using the individual SNP and haploblock approaches indicated that genomic predictions could be improved using LD-based haploblocks as explanatory variables in prediction models, in some cases. When the prediction reliabilities for both the BLUP and Bayesian mixture models were compared (Figures 2, 3 and 4) and the p-values of Hotelling’s test were analysed, the results provided strong statistical evidence that using haploblocks built under the threshold *D*^′^≥0.45 increased prediction accuracy for all three traits tested, in an analysis of the three traits all-together. Moreover, these results were achieved when the haploblocks were used along with the non-blocked SNPs.

The prediction reliabilities (Figures 2, 3 and 4) implies that the haploblock approach improved the predictability for traits with high heritability, for example, the milk protein trait (*h*^2^=0.39, for which the reliabilities obtained by the models using the haploblock approach were clearly superior to the reliabilities obtained using the individual SNP approach. For traits with low heritability, such as fertility and mastitis (both with *h*^2^=0.04), any benefits of using the haploblock approach could not be identified based on a simple graphical overview of the results.

When the Hotelling’s test was applied, the initial inferences based on the graphs were confirmed, and in every scenario the haploblock approach was shown to provide superior predictability of milk protein compared with the individual SNPs. For fertility, an improvement in predictability using the haploblocks was confirmed; however, the most significant improvement was seen when a medium amount of LD was considered to build the haploblocks, *i.e.* when *D*^′^≥0.45. For mastitis no significant improvement in prediction accuracy could be found using the haploblocks rather than the individual SNP approach.

For both the BLUP and mixture models, the haploblocks built considering *D*^′^≥0.45 with the non-blocked SNPs, as explanatory variables, resulted in greater reliability than the individual SNP approach for milk protein, fertility and mastitis, and this result was statistically significant (Hotelling’s test). Although this threshold did not result in the highest prediction reliability for all traits, it presented the best results for mastitis, which was the most unstable trait to predict. Hence, this particular scenario may be useful to improve the predictive ability of different dairy traits. Furthermore, the bulls selected by haploblocks models were very consistent when using different *D*^′^ thresholds. Hence, the appointment of a possible optimal *D*^′^ threshold to build haploblocks was mostly based on the analysis of the prediction reliabilities and Hotelling’s tests obtained for the three traits all-together.

Comparisons of the BLUP and Bayesian mixture models showed statistically significant differences mainly for milk protein, which is consistent with previously reported results for protein and fertility [6, 31]. Gao et al. [31] found that the advantage of the mixture model over the BLUP was more profound with weak relationships between training and data sets, and the authors argued that the mixture model captured LD between markers and a QTL more efficiently than the BLUP model.

In this work, the main aim was to use haploblocks to perform genomic prediction, based on the assumption that haplotypes are in stronger LD with the causative mutation than are the individual SNPs, because a QTL in weak LD with any individual marker may be in strong LD with a multi-marker haplotype. In addition, haplotypes can better capture mutations in more than one loci. Allele frequencies may change very little when a mutation occurs at a locus, but the frequencies of variants in a haplotype are more likely to change when mutations occur in one or more loci of a haploblock [11]. Therefore, haplotypes may be better able to identify a QTL region than individual SNPs.

A secondary focus of this study was haploblock design and the need to reduce the number of variables in HD marker data without loss of information. When haploblocks are designed based on LD between HD markers, they tend to reduce the amount of variants automatically, because the combination of SNPs within a haploblock is not random. Haploblocks defined according to the LD usually reflect the characteristics of the genome better than haploblocks artificially outlined by a fixed number of SNPs. The variable reduction provides as a desired consequence, reduction of the computing time for the genomic prediction models. This reduction in computing time is proportional to the reduction in the number of variables.

Until now, the majority of studies on the use of haplotypes for genomic prediction have used simulated and moderate density data and not HD data [15–19]. In these studies, the number of SNPs used to outline haploblocks was arbitrarily defined, which generated artificial haplotypes and their variants, and as a result, there was neither focus on the efficient use of the properties of the genome to define haploblocks, nor was there a need to reduce the number of variables for the genomic prediction models. In [20], haplotypes based on HD marker data are defined using Beagle [21], however fixedly defined as the first locus out of ten consecutive loci in genomic evaluation. Although the results obtained with the method described by [20] indicate improvement in genomic prediction, it is not possible to distinguish if the haplotypes are indeed the cause of higher prediction reliabilities, since the approach also involves a multi-breed panel and the use of cows in the reference population.

In the present study, the use of LD to define haploblocks helped in determining the location and the length (number of SNPs) of the haploblocks. LD can quantify non-random associations between any two loci, and is a very good measure to use for building haploblocks using the properties of the genome. When a defined minimum LD between any two loci is used to select a group of adjacent SNPs to outline a haploblock, the number of variants of the haploblock will be reduced compared with no LD between the markers, when random associations between the SNPs will produce many more variants per haploblock.

When LD is used to define haploblocks, data density also needs to be considered. For example, HD data (e.g. 777 *k*) will have higher LD measures for adjacent SNPs than medium density data (e.g. 54 *k*), meaning that in HD marker data, more haploblocks, containing more markers within a haploblock, are likely to be built compared with the haploblocks in 54 *k* data. Similar results can be expected when even higher density data (*e.g.* whole genome sequences) is used. Thus, haploblocks may reduce the number of variables in marker data that are denser than HD data, while keeping all the SNP information that the data contains.

In this study, six different *D*^′^ thresholds were evaluated and compared in BLUP and Bayesian mixture models. The results indicated that the optimal threshold for the haploblock approach was *D*^′^≥0.45 in both models. When this threshold setting was applied, the models displayed better prediction accuracy for all three traits studied compared with the individual SNP approach. The results indicated that choosing the optimal threshold to define haploblocks was important for obtaining accurate predictions, especially for the low heritability traits (fertility and mastitis).

Haploblocks are built for each chromosome separately; therefore, when extreme *D*^′^ thresholds are set (zero and 1) *D*^′^≥0 means a whole chromosome is selected as a haploblock and *D*^′^≥1 means each individual SNP is a haploblock.

## Conclusion

The statistical methods used in this work to build the LD-based haploblocks from HD data produced a better prediction accuracy than the individual SNP approaches for some traits, that are widely used in genomic prediction of economically important traits in dairy cattle. The benefit of using the haploblock approach in genomic prediction models was much larger for milk protein compared with its benefit for fertility or mastitis. The identified evidence was quiet strong that building haploblocks using a *D*^′^≥0.45 threshold produced an optimal set of variables for all three traits tested. The choice of this suggested *D*^′^ threshold was made not only based on the amount of increase in prediction reliability for each trait. It was decided for 0.45 due to the fact that it was the threshold that provided increase for all three traits, when compared to the individual SNPs approach. Furthermore, 0.45 resulted in the greatest increase in prediction reliability for mastitis, which was the most unstable in prediction reliability with the change of the *D*^′^ threshold. There was a desire to appoint one *D*^′^ threshold that could benefit the prediction of the three traits simultaneously, and 0.45 fulfilled that.This method should be explored further in future genomic predictions of dairy-related traits.

The results reported here will be relevant for genomic selection in animal breeding because HD marker data is now widely available, and even denser marker data is likely to become available soon. The use of LD-based haploblocks as explanatory variables for genomic prediction models is likely to increase in the future. This study has shown that to achieve significantly better prediction accuracy, it is important to determine an optimal *D*^′^ threshold to build haploblocks from HD marker data.
